# The repercussions of burnout among health care professionals in medical oncology in tunisia

**DOI:** 10.1192/j.eurpsy.2021.1149

**Published:** 2021-08-13

**Authors:** A. Daldoul, W. Khechin, W. Krir, F. Ezzairi, H. Kefi, S. Zaied, S. Ben Ahmed

**Affiliations:** 1 Medical Oncology, Fattouma Bourguiba University Hospital, Monastir, Tunisia; 2 Psychiatry, Military Hospital, Tunis, Tunisia; 3 Medical Oncology, University Hospital, sousse, Tunisia

**Keywords:** burn out, oncology

## Abstract

**Introduction:**

Freudenberger was the first to define burnout as a feeling of helplessness and guilt, as well as boredom and disinterest.

**Objectives:**

Our study aimed to analyze functional complaints and the behavior of healthcare professionals in this area associated with this syndrome.

**Methods:**

This was a cross sectional study including health care professionals in medical oncology working in public hospitals in Tunisia. It was carried out from 15 January 2019 to 15 June 2019. Health professionals were asked to answer the Maslach –Burnout Inventory Test.

**Results:**

The average age was 34 years ± 6.7. Burn-out was found in 15 of the participants, (21%). In our population, a high emotional exhaustion score was significantly associated with its repercussions: Sadness, Blockage, sleep disturbances, unexplained pain, Epigastralgia / fatigue, Addictive behavior ; avoidance behavior, repercussions on the relationship with those around them, desire for a transfer, regret for choosing a profession, suicidal thoughts, absenteeism and smoking. A high depersonalization score was significantly associated with several functional and behavior complaints, in particular: irritability, anger, feeling of indifference, guilt, unexplained pain, decreased performance, suicidal thoughts. A low personal achievement score was significantly associated with psychotropic drug use. Global burn-out was significantly associated with feeling of blockage, guilt, unexplained pain, epigastralgia and fatigue, addictive behaviors and avoidance behaviors as well as suicidal thoughts, absenteeism and consumption of psychotropic drugs.

**Conclusions:**

Through its impact on professionals, burnout in medical oncology represents a major threat to the quality of care and the survival of institutions.

